# A spatial–temporal graph deep learning model for urban flood nowcasting leveraging heterogeneous community features

**DOI:** 10.1038/s41598-023-32548-x

**Published:** 2023-04-25

**Authors:** Hamed Farahmand, Yuanchang Xu, Ali Mostafavi

**Affiliations:** 1 Zachry Department of Civil and Environmental Engineering, Texas A&M University, College Station, TX USA; 2 Department of Computer Science and Computer Engineering, Texas A&M University, College Station, TX USA

**Keywords:** Natural hazards, Civil engineering

## Abstract

Flood nowcasting refers to near-future prediction of flood status as an extreme weather event unfolds to enhance situational awareness. The objective of this study was to adopt and test a novel structured deep-learning model for urban flood nowcasting by integrating physics-based and human-sensed features. We present a new computational modeling framework including an attention-based spatial–temporal graph convolution network (ASTGCN) model and different streams of data that are collected in real-time, preprocessed, and fed into the model to consider spatial and temporal information and dependencies that improve flood nowcasting. The novelty of the computational modeling framework is threefold: first, the model is capable of considering spatial and temporal dependencies in inundation propagation thanks to the spatial and temporal graph convolutional modules; second, it enables capturing the influence of heterogeneous temporal data streams that can signal flooding status, including physics-based features (e.g., rainfall intensity and water elevation) and human-sensed data (e.g., residents’ flood reports and fluctuations of human activity) on flood nowcasting. Third, its attention mechanism enables the model to direct its focus to the most influential features that vary dynamically and influence the flood nowcasting. We show the application of the modeling framework in the context of Harris County, Texas, as the study area and 2017 Hurricane Harvey as the flood event. Three categories of features are used for nowcasting the extent of flood inundation in different census tracts: (i) static features that capture spatial characteristics of various locations and influence their flood status similarity, (ii) physics-based dynamic features that capture changes in hydrodynamic variables, and (iii) heterogeneous human-sensed dynamic features that capture various aspects of residents’ activities that can provide information regarding flood status. Results indicate that the ASTGCN model provides superior performance for nowcasting of urban flood inundation at the census-tract level, with precision 0.808 and recall 0.891, which shows the model performs better compared with other state-of-the-art models. Moreover, ASTGCN model performance improves when heterogeneous dynamic features are added into the model that solely relies on physics-based features, which demonstrates the promise of using heterogenous human-sensed data for flood nowcasting. Given the results of the comparisons of the models, the proposed modeling framework has the potential to be more investigated when more data of historical events are available in order to develop a predictive tool to provide community responders with an enhanced prediction of the flood inundation during urban flood.

## Introduction

### Background

Flood nowcasting is a process by which areas at imminent risk of inundation can be identified using the spatial and temporal features that convey information regarding current flooding status. As extreme weather events accompanied by heavy precipitation occur more frequently, causing catastrophic flood events, flood nowcasting has become an essential capability for communities to better respond to the impacts of these events^1^. Flood nowcasting enables predictive flood monitoring, the ability to anticipate imminent flood risks and impacts and situational awareness as an extreme weather event unfolds^2^. Departing from the standard flood monitoring approaches using hydraulic and hydrological (H&H) models that predict flood inundation levels for hazard mitigation and infrastructure improvements prior to flood events^3,4^, flood nowcasting focuses on near-future prediction (e.g., next few hours) of spatial and temporal flood status based on the current status of flooding. The traditional approaches for flood monitoring^5^ do not provide certain essential information (e.g., what areas will be inundated within the next few hours). Nowcasting will enable public officials, emergency managers, responders, and residents to better tailor decisions and actions by enhancing situational awareness during response and recovery^6^. Hence, urban flood nowcasting facilitates identifying areas that will require emergency aid in hours immediately ahead and areas that need issuance of evacuation notices due to the high risk of flood inundation. This forewarning is critical for reducing the adverse impacts of flood events. It also facilitates taking proper managerial actions to control flood inundation using hydrological infrastructures, such as flood gates and pumps^7–9^. The main approach for sensing flood status is the use of rainfall and stream gauges; however, due to cost and maintenance limitations, the number of these physical sensors is limited, which affects proper observability of flood status^10^, and hence, flood nowcasting. New techniques for enhancing situation awareness and emergency response actions leverage heterogeneous community-scale datasets (including both physical sensors and crowdsourced data) in advance to provide the predictive capability to infer the flooding status for the near future in spatial units (e.g., zip code, census tract, and neighborhood), information^11–13^.

Multiple studies have been conducted to develop predictive tools using a wide range of physics-based features and quantitative techniques. Conventionally, H&H simulation models are used for predicting the extent of flooding in urban areas using geomorphological hydrodynamic features to estimate water depth in urban areas^14,15^. These models often rely on the data collected from rainfall and flood gauges to provide an estimate of the spatial extent of flood propagation^6,16^. Despite their satisfactory accuracy and predictive performance, extensive computational cost and the sparsity of the reliable hydrological data in urban areas limit the existing physics-based H&H models^17–19^ for providing near-future estimation for spatial–temporal propagation^20^. To complement the standard models, recent studies tested data-driven models based on harnessing data sources, such as satellite images, crowdsourced data, and remote-sensing data, that can help estimate flood status in near future timeframes^3,21–24^. Also, a growing number of researchers have used the predictive capability of various machine learning (ML) models for flood predictive monitoring^25–30^. These models can include more community features than tradition models to forecast flood status, which facilitates capturing the large number of heterogeneous community features needed for flood nowcasting^31,32^.

In the following sections, we review the state-of-the-art in application of deep-learning models for flood nowcasting to identify gaps in the existing literature. We focus particularly on two major gaps: (1) the absence of a model architecture that enables capturing spatial and temporal dependencies in flood propagation and dynamically identifying influential features, and (2) limited efforts for integrating human sensing as an approach for collecting and extracting valuable temporal and spatial data. We also review the use of heterogenous human-sensed data as a supplement for flood nowcasting to show the gap in the knowledge regarding the proper use of such data for improving the urban flood nowcasting models. Accordingly, we adopt a model proposed by Guo et al.^33^ and test a novel graph-based deep-learning models that enable capturing spatial dependencies, as well as heterogeneous human-sensed features in flood propagation. We demonstrate the application of the proposed model in the context of the 2017 Hurricane Harvey flooding in Harris County, Texas.

## Related works

### Deep learning for flood nowcasting

Advances in machine learning techniques are responsible for the emergence of deep learning (DL), a sub-domain of ML that employs deep artificial neural network architectures and gradient descent algorithms for yielding more robust and computationally efficient predictive models^34–38^. Deep neural networks have been increasingly used for tasks that support flood predictive monitoring, such as flood depth mapping and flood detection. Multiple studies have applied DL techniques to improve the predictive performance of physics-based flood nowcasting models. For example, a convolutional neural networks (CNN) have been used in combination with conditional generative adversarial networks (cGAN) to improve the performance of physics-based flood forecast models^39^. In addition, a combined empirical mode decomposition (EMD) algorithm and encoder-decoder long short-term memory (En-De-LSTM) architecture have proved to yield a better prediction of peak flow values of streams during floods^40^. Recent data-driven models rely purely on the capability of DL models for flood prediction. For example, streamflow prediction using an integration of stacked autoencoders (SAE) and back propagation neural networks (BPNN) show higher accuracy compared with other tested ML models^41^. Also, Gated Recurrent Units (GRU)-based network architecture has been utilized for predicting the time series of stream sensors used for flood monitoring^42^. In a recent work by Dong et al.^39^, a Fast GRNN-FCN (fast, accurate, stable, and tiny gated recurrent neural network-fully convolutional network) was proposed for forecasting the water level in channel network sensors to provide flood signals in flood control network^43^. While the use of DL models for flood prediction is becoming prevalent in the literature and practice, the current research trends lack a computational data-driven modeling framework that enables a near-future prediction of flood status in spatial blocks (e.g., census tracts or zip codes). This gap is due mainly to: (1) inability of the existing models to capture the spatial interdependencies; (2) limitations in extracting features that provide indication of flood status in spatial blocks (due mainly to a limited number of physical sensors). The inability to predict near-future flood status in spatial blocks is a major hindrance to flood nowcasting. To address this gap, in this study, we propose a spatial–temporal graph deep-learning model.

Incorporating attention mechanisms into spatial–temporal deep-learning models for flood prediction elicits superior results compared to other state-of-the-art model architecture, and also improves interpretability of the model results^44^. These studies often leverage the ability of different DL models for time-series forecasting and early warning detection utilizing sensors that collect rainfall and streamflow data. Nevertheless, most recent studies have: (1) employed DL architectures that enable incorporating spatial correlation, and (2) created DL architectures that enable more feature incorporation. For spatial correlation, graph neural networks can capture the spatial similarity of model units^45^ while an attention mechanism that enables the model to focus on the characteristic data when processing large numbers of features^46^ and enable use of heterogeneous data to provide reliable prediction in urban units. In the next sections, we discuss the application of graph neural networks for spatial and temporal prediction as well as using heterogeneous data in flood predictive monitoring, which form the points of departure for this study.

Other than the deep learning models for flood nowcasting, coupled hydatic and hydrologic models that use multiple GPUs for forecasting urban flooding in real time r near real time have gain attention in the literature. The advantage of these models is that they can provide accurate flood maps using various surface and social properties as well as hydraulic and hydrologic properties. For example, coupled hydrologic–hydraulic model (HiResFlood-UCI)^47^ for flash flood modeling has been developed to increase the efficacy of the hydraulic modeling to produce high-resolution flood maps. A fully coupled hydrologic-hydraulic modeling framework was also developed for flood prediction and modeling for both riverbank and overland inundation, which shows superior performance^48^. To enhance the performance of these models and reducing uncertainty, different approaches such as assimilation of satellite-based synthetic aperture radar (SAR) observations into the coupled mode have been proposed^49^. Besides, techniques such as remote-sensing have also been used for calibrating these models to improve their precision for flood detection^50^.

### Graph neural networks for spatial–temporal prediction

Graph neural networks generalize convolution to data in a graph structure^51^. With their superior capability to characterize spatial and temporal dependencies for time-series predictions, Graph Convolutional Networks (GCNs) characterize networked data with spatial and temporal dependencies for time-series prediction using spatial and temporal convolutions. These models (referred to as spatio-temporal graph convolutional network (STGCN) models) are used for prediction problems such as traffic flow prediction^52,53^, disease diagnosis^54^, bike-demand prediction^55^, point-of-interest (POI) recommendation^53^, pedestrian flow prediction^56^, trajectory prediction^57^, and road network flood inundation prediction^2^. STGCN model architectures have been developed based on the problem characteristics. For example, dual-channel based graph convolutional networks (DC-STGCN) consider both daily and weekly correlation of the traffic data^58^. Discriminative spatio-temporal graph convolutional network (DSTGCN) were used for action recognition in to inner-class action distribution^59^. Wang et al.^5^ developed an auto-STGCN algorithm that facilitates the detection of the optimal STGCNs models automatically using a reinforcement learning technique^60^. An attention mechanism allows DL models to focus more on the useful parts of features^46^. In graph neural networks, the attention mechanism allows the model to learn a dynamic and adaptive combination of the adjacency matrices and select the most relevant information^61^. Attention-based GCNs adaptively capture dynamic spatial and temporal correlation of heterogeneous data and its interpretability power^33^. The combination of attention mechanism and STGCN structure, therefore, could provide a powerful testbed for problems in which heterogeneous features with complex spatial and temporal correlation exist. The application of attention-based STGCNs in the literature, however, is limited to traffic flow prediction^33^. Because of the characteristics of the urban flood nowcasting problem, attention-based STGCNs may provide models that could account for spatial interdependencies, as well as for the temporal correlations among features related to flood inundation status.

### Heterogeneous human-sensed data for flood nowcasting

To complement the information sensed by physical sensors, other sources of data with distinct levels of reliability, aggregation, and the need for preprocessing have been tested in recent studies^31^. Satellite images, drone-recorded videos and images, and images captured by other cameras provide reliable information; however, limitations of data acquisition and challenges in data processing restrict extensive use of such data for flood predictive monitoring and vulnerability assessment^62–65^. Blumberg et al.^1^ employed hurricane-related photos provided by volunteers to simulate flood inundation during 2012nHurricane Sandy in Hoboken and Jersey City, New Jersey. On the other hand, human-sensed crowdsourced data have become more available in different formats that can provide geo-located information regarding the flood status in a timely manner. For example, studies have analyzed anonymized social media content using ML and DL techniques and employed the extracted information for enhancing flood situational awareness^32,66–68^. In another study example, Huang et al.^69^ integrated tweet data gathered by remote sensing and river water gauges to improve near real-time flood inundation maps. Nevertheless, the tweet activity data has also proven to expedite the detection of flood inundation and flood-related events when combined with satellite flood signals^70^. However, there are limitations in terms of content analysis and ensuring the credibility of the extracted information from social media^71^. Furthermore, social media data might be biased by factors such as distance to impacted areas, the popularity of the user, and demographic characteristics of users^72^. Recently, the digital trace of human activities (such as cellphone and location-based data) has also been deployed for flood prediction. The rationale is that the changes in the level of human activity and the concentration of human activity can indicate signals regarding flood status^73^. The combined use of different sources of data—physical flood sensors data, crowdsourced social media data, and telemetry-based human activity data provides opportunities to gather a more extensive set of indicators related to flood status for use in flood predictive monitoring^74^. Integrating such heterogeneous data requires a modeling framework that is able to recognize and focus on key data features. The attention-based STGCN model proposed in this study enables leveraging heterogeneous datasets to capture features related to flood inundation status for flood nowcasting^33^.

### Point of departure

The review of the current state of the art shows two gaps in the knowledge for urban flood nowcasting: (1) the absence of a deep-learning structure that combines attention mechanism and graph-based convolutional network structure for extracting information from heterogeneous features with complex spatial and temporal correlation; and (2) the lack of a proper flood nowcasting modeling framework for integrating heterogeneous human-sensed features that can carry valuable flood-related information along with the physical sensor data. Recognizing these gaps, this study presents a deep-learning modeling framework including an adopted attention-based spatial–temporal graph convolution network (ASTGCN) model and streams of data that could be collected as a flood event unfolds, preprocessed, and fed into the prediction model to consider spatial and temporal features as well as dependencies in order to enable reliable urban flood nowcasting. The proposed model was tested in the context of flooding caused by the 2017 Hurricane Harvey in Harris County, Texas. The model performance and its implications for flood nowcasting, as well as enhancing situation awareness, are discussed. The novelty of this study is the creation a framework that addresses major limitations in the application of data-driven techniques for flood nowcasting by (1) focusing on graph-based architectures that enable co-location dependency between urban units for considering the spatial aspect of flood propagation, (2) identifying and processing various heterogeneous physics-based and human-sensed data that carry information for inferring flood status in spatial units, and (3) utilizing an attention-based time-series forecasting architecture for considering the temporal aspect of flood prediction and focusing on information with higher importance when processing large amounts of heterogeneous features.

## Methods

### Problem definition and abstraction

In this study, we model the study area as a network of census tracts to capture the spatial interdependence in urban flood propagation and recession. We used the census tract as the spatial unit for various reasons: its scale is neither so coarse as to lose the resolution nor so fine as to lose observability of flood status due to missing data. This makes the census tract a suitable spatial scale for aggregating and interpolating both human-sensed data and physics-based data while maintaining data accuracy and keeping it informative for flood nowcasting. Next, the purpose of any urban prediction model is to provide emergency managers and people with actionable data. Therefore, the alignment of spatial units of the outputs with the administrative boundaries make the results more insightful and valuable for decision makers. In addition, one issue that is associated with the use of human-sensed data is that these data are biased toward highly populated areas. Therefore, if the spatial unit is smaller in the locations with higher population, the bias is alleviated to some extent. Finally, demographic data is available for administrative boundaries with proper accuracy. Therefore, future research can focus on the issues related to the associated between model performance in areas with different demographic characteristics to investigate crucial aspects of the model such as fairness and demographic biases.

**Figure 1:**
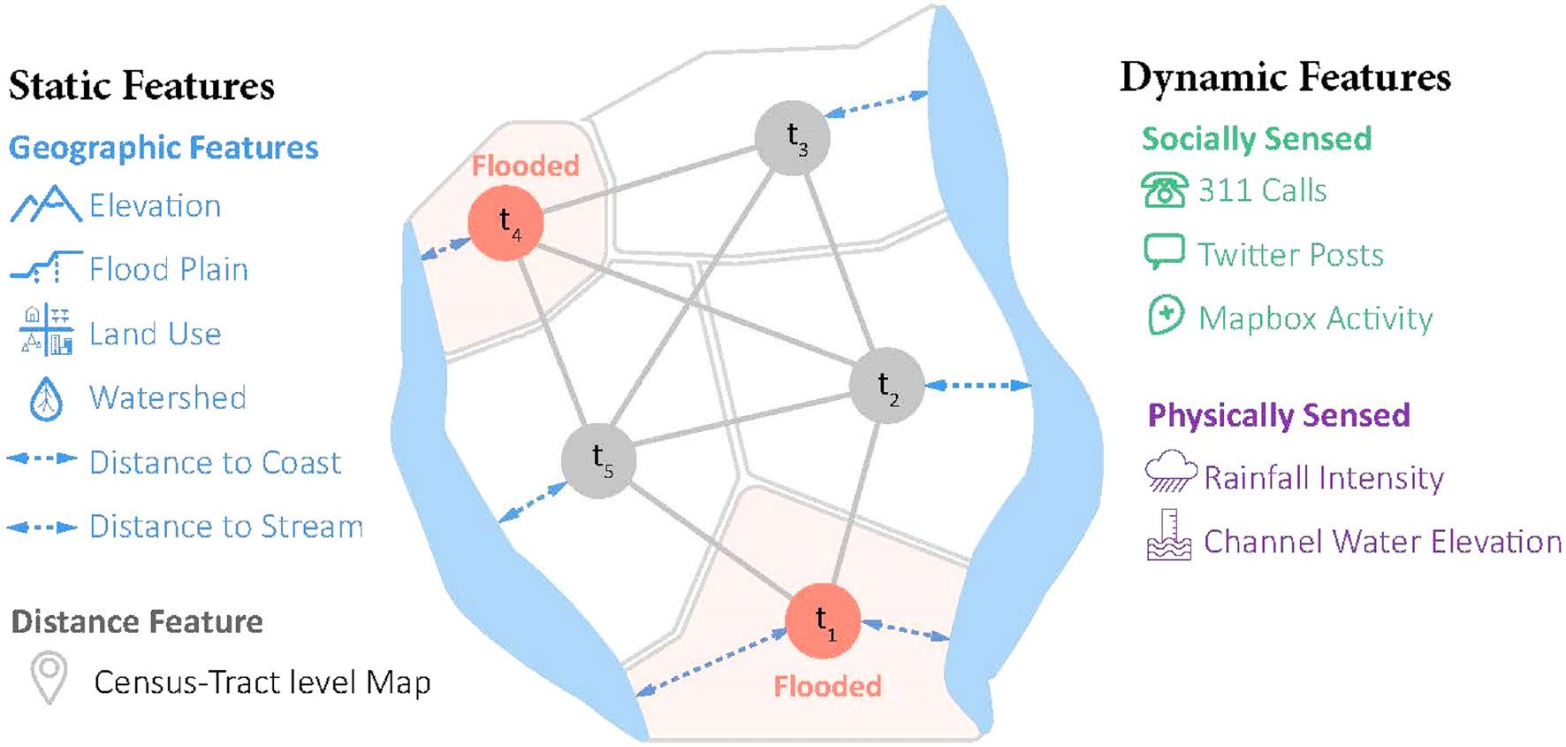
Schematic representation of the problem abstraction; the study area is modeled as a graph; static features and distance are used to determine weights, and physics-based and human-sensed dynamic features are used for predicting the extent of flooding.

We created an undirected graph $G=(V,E,A)$, where $V$ is the set of $N$ nodes, each representing a census tract in the study area; set $E$ includes edges in graph $G$ that represent the connection between different nodes; and matrix ${A}_{N\times N}$ is the adjacency matrix of graph $G$. Entries of matrix $A$ are determined based on the proximity and the extent to which two census tracts have similar features that potentially influence their flooding status. Therefore, matrix $A$ is built upon the distance between census tracts and a set of static features, such as elevation, land use, and distance to stream, that impact the flooding status of particular areas^75^. At each timestep, each node in the graph $G$ holds a vector of temporal features (more discussion about the features are provided in the next section) that contain information that is used as the model input for nowcasting flood in the model. These temporal features capture various physics-based and human-sensed data inputs that are aggregated and preprocessed into the same sampling frequency. Figure 1 shows a schematic representation of the graph model, as well as static and dynamic features that are used for feeding the model for flood nowcasting.

### Overview of the model development and evaluation

**Figure 2:**
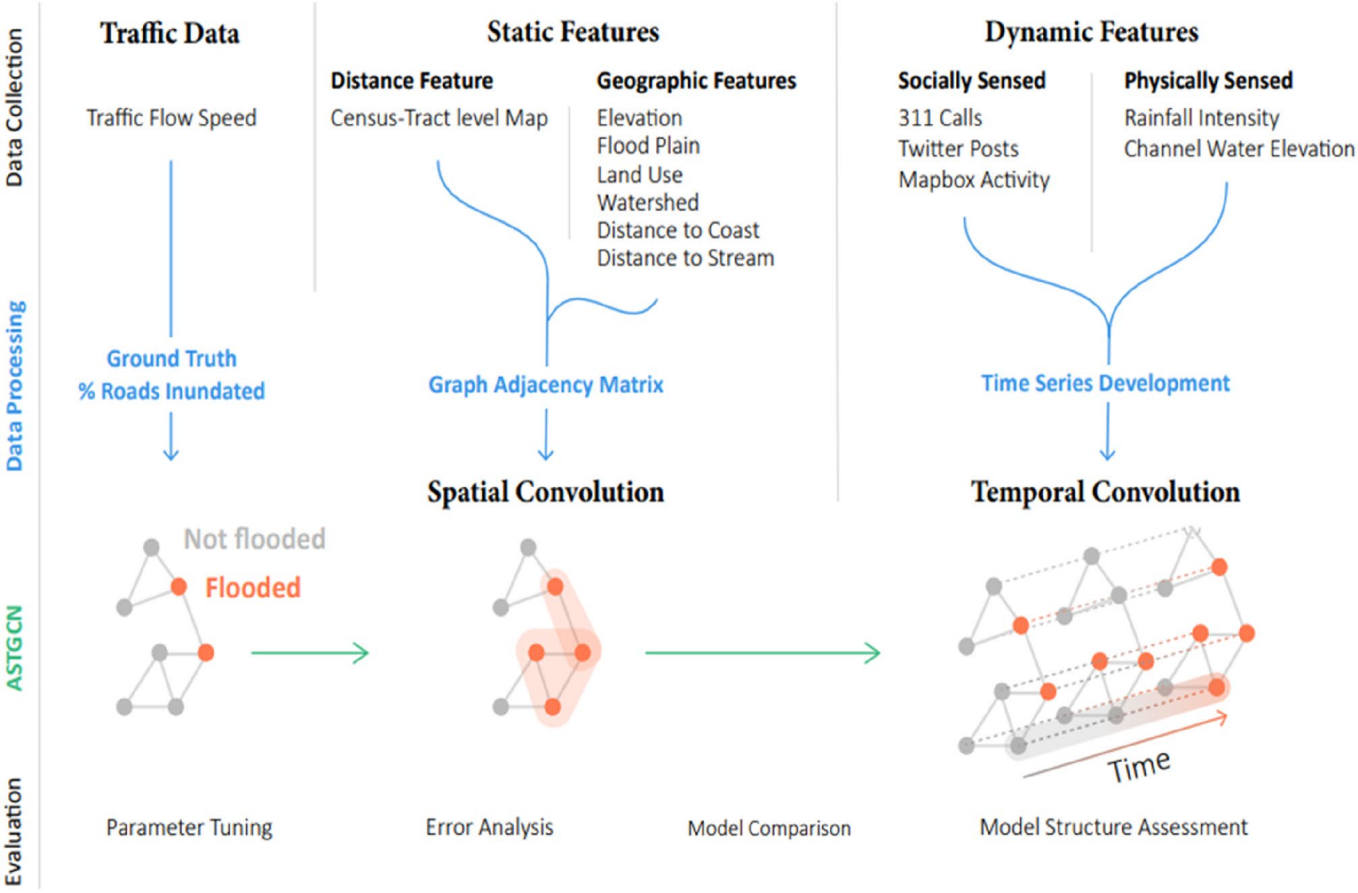
Overview of the model framework, including steps for collecting and preprocessing ground truth and features, developing the ASTGCN model architecture, and evaluating the performance of the model.

Figure 2 shows the overview of the steps for the development and evaluation of the model. Overall, the design plan for the study involves four steps^76,77^: data collection, data preprocessing, model development, and model evaluation. First, we present the data used for the development of the model. The data includes ground truth data: static features, which represent the dependency between flooding status of different areas in the adjacency matrix; and dynamic features that provide indications of temporal propagation and recession of urban flooding in each census tract. We also elaborate on the data preprocessing needed for the preparation of static features and the construction of time series of the dynamic features. Then we present the model architecture and mechanisms used in the DL model for urban flood nowcasting. Finally, we discuss the performance evaluation metrics of the model, parameter tuning for optimizing the model performance, and comparison of the model performance with other state-of-the-art models.

### Data collection and preprocessing

#### Ground truth

We used traffic condition data for 19,712 road segments in Harris County provided by the private company INRIX as proxies to determine if a certain road section was flooded. INRIX collects location-based data from both sensors and vehicles. The INRIX traffic data contains the average traffic speed of each road segment at 5-min intervals and their corresponding historical average traffic speed. Each road segment’s identification information, such as name, geographic locations defining its head and end coordinates, and length, is also available from the INRIX data set. Previous research shows that road segments flooded due to Hurricane Harvey can be indicated by detecting the road segments with NULL values for their average traffic speed^20^. We filtered the road segments with $speed limit \ge 30$ mph to only account for the main roads in inundation estimation. We found that this data division helps to reduce the data imbalance problem by capturing more flooded segments. The filtered data is used to determine the percentage of roads flooded as the indicator of the flood extent in census tracts. To do so, we characterized the flood status of each census tract as the ratio of flooded roads to the total number of roads.

#### Static features

Static features were used to develop the adjacency matrix and assign weights of connection between nodes in the graph model. We developed the adjacency matrix primarily based on the distance between the centroids of census tracts. In addition, we incorporated the impact of six static features (five features in the Table 1 and physical distance) that characterize flood propagation in an area in the adjacency matrix. The rationale is that nodes that have similar static features would have similar flood propagation behavior. Table 1 shows static features including floodplain, land use, watershed, distance to coast and distance to stream and the description of how they are calculated. These features were collected for each census tract. For the features available for each point, the value of the centroid of the census tract is considered. Elevation from the sea level was calculated using the digital elevation model (DEM) of the study area. Distance to Galveston coast and distance to closest main streamflow were calculated by mapping the study area and the streams that discharge stormwater from the area into Galveston Bay. Moreover, we coded 22 watersheds within the study area, and each census tract was associated with the watershed within which its centroid falls. Similarly, we mapped the 100-year floodplain and determined whether the centroid of the census tract falls inside the floodplain. The resulting binary variable was then used as a static feature. Finally, we used the land-use map of Harris County and determined the ratio of residential area to total land area as a feature that is a determinant of the land properties.

**Table 1:** Static and dynamic features used for urban flood nowcasting.

Influencing factor	Feature
Static feature	
Floodplain	Whether or not the area is inside the 100-year floodplain
Land use	Percentage of the residential area
Watershed	The watershed that the area falls inside it
Distance to coast	Distance to Galveston coast
Distance to stream	Distance to the closest main stream flow
Dynamic feature	
Physics-based features	
Short-term rainfall intensity	Estimated accumulated rainfall in past 2 h
Long-term rainfall intensity	Estimated accumulated rainfall in past 24 h
Water elevation	Estimated ratio of water level to the flooding threshold, based on average readings of two closest channels
Human-sensed features	
Flood reports	Number of reported flooding in the neighborhood through 3–1-1 platform
Social media activity	Number of flood-related filtered tweets
Human activity	Activity index of telemetry-based digital trace of human activity

As a summary, ground truth is used as the dependent variable in the classification in the model. In addition, in case of static and dynamic features, the rationale for selecting static features is to capture similarities between spatial units of analysis to assign weights between them. The degree of influence was determined by testing different weights for the influence of static features versus physical distance. For dynamic features, the rationale for selecting features is (1) ability to provide temporal flood-related information and (2) availability of the temporal data with proper spatial resolution. The degree of influence of dynamic features were tested by developing different models that use different data inputs and investigating the performance of each model.

#### Dynamic features

**Figure 3:**
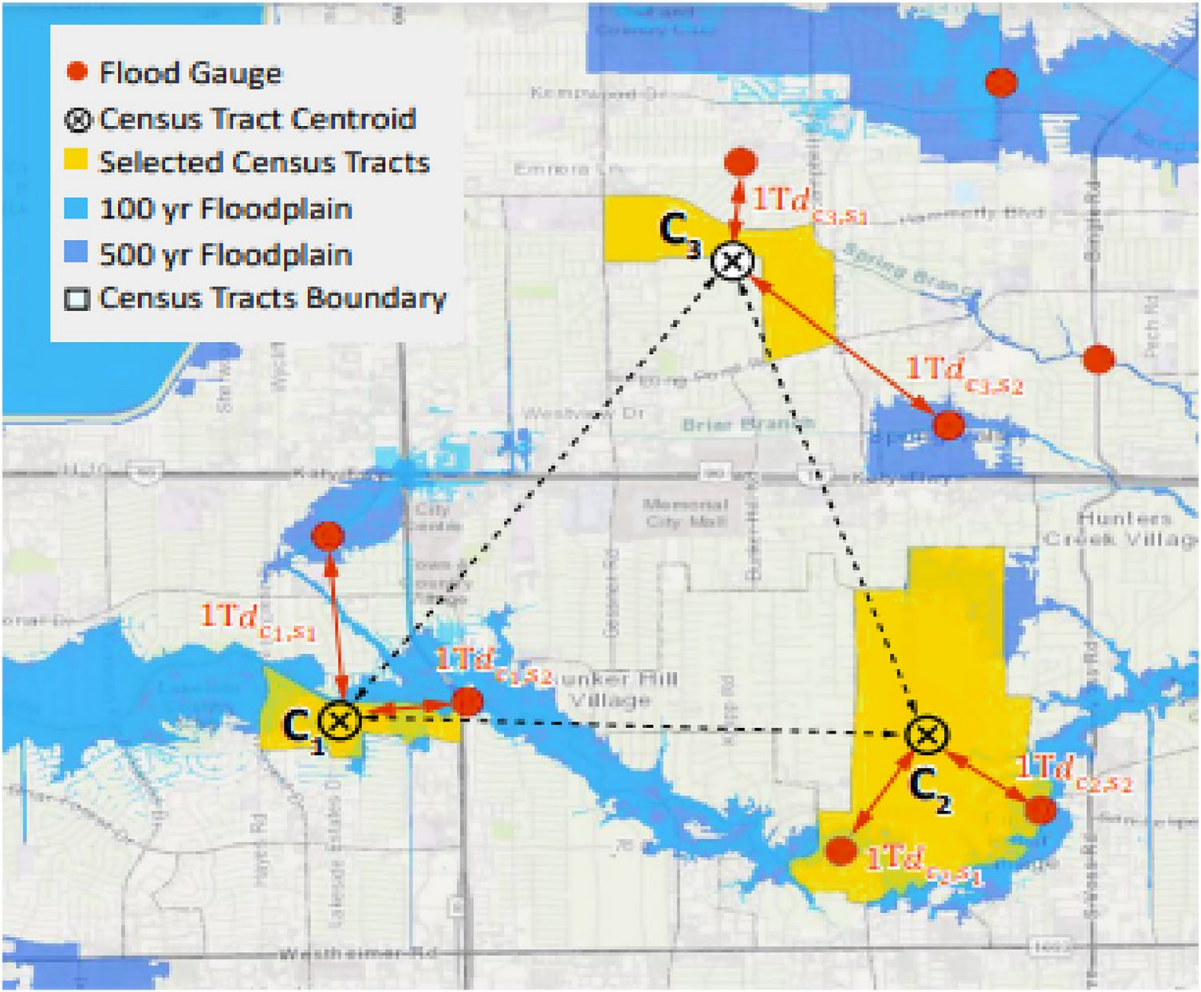
Schematic illustration of calculating census tracts’ distances and weights of two closest flood gauges as inputs to the census tract physic-based features.

Dynamic features capture temporal changes that can indicate the flood propagation and can be used by the model for flood nowcasting. We considered both physics-based and human-sensed features. For physics-based features, we used the data recorded by the 175 flood gauge stations in Harris County. These flood gauge stations are located on the main channels and bayous to provide residents with timely information on rainfall accumulation and water elevation in the stream^78^. We collected the rainfall and stream elevation from the official website of Harris County Flood Control District^78^. We constructed three time series for each census tract based on the flood gauge data, including short-term rainfall intensity, long-term flood intensity, and water elevation. For short-term rainfall intensity, we used the accumulated rainfall in the past 2 h recorded by the flood gauge (Table 1). For long-term rainfall intensity, we used the accumulated rainfall in the past 24 h recorded by the flood gauges. Also, we used the ratio of recorded water elevation to the threshold elevation of flooding in each flood gauge as the water elevation indicator. It should be noted that the frequency of readings of rainfall and water elevation varies across time; in such cases, we performed interpolation and extrapolation to extract the value of the time series based on the available readings. The number of flood gauges is fewer than the number of census tracts; therefore, we used the weighted average of readings of the two closest flood gauges to determine measurements for each census tract. Weights are proportional to the inverse of the distance between the centroid of the census tract and the flood gauge. Figure 3 illustrates the process for determining physics-based features for each census tract based on the flood gauge data.

Physics-based features provide a reliable source for indicators needed for flood nowcasting; however, due to limitations such as sparsity of data points and lack of sufficient data (limited number of physical sensors) for inferring flood status in near future, we used a number of human-sensed data types to supplement the data needed for flood nowcasting. We used three different types of human-sensed data (Table 1): records of 3–1–1 flood reports, Twitter activity, and the telemetry-based digital trace of human activity. We collected 4275 flood-related 3–1–1 reports for the study period from the official website of the City of Houston^79^. Then, we filtered reports based on report type so that only reports that indicated flooding were included. More 3–1-1 flood reports during a certain timeframe in an area indicate a higher risk of flooding^75^; thus, we spatially aggregated the number of reports in each timestep and created a time series showing the number of floods reported through the 3–1–1 platform for each census tract. Social media platforms are another means by which people disseminate information regarding flooding in near real-time. Hence, the relevant data collected from social media can improve flood nowcasting. We incorporated flood-related information posted by Twitter users as an input for our flood nowcasting model. The geotagging feature of Twitter links tweets with accurate longitude and latitude of the location from which tweets originate ^80^. Although a small percentage of tweets have geotagged, this small percentage generates thousands of tweets that provide reliable insights into flood status, especially areas lacking physical sensors. To examine social media attention, we used collected tweets for the study time period (August 25, 2017, to September 2, 2017) in 84 super-neighborhoods in Houston. Twitter PowerTrack API (application programming interface) was used for collecting the 29,256 geotagged tweets during the study time period. Two filters were applied to ensure the relevance of the tweets. The first filter identifies the tweets, whose geotags like in our predefined bounding boxes, posted by the users whose profiles show their location Harris County. The second filter was the keywords (i.e., the names and abbreviations of the areas) that identify the tweets specifically related to the study area^71^. Regarding the use of Twitter data, it should be noted that multiple research studies show that crowdsourced data such as Twitter data are subject to various biases such as population bias, spatial bias, and sample bias^80,81^. Particularly, there are studies that investigate the biases in geotagged tweets for Hurricane Harvey^75^. However, one of the potential promises of the model developed in this study is to alleviate the biases in the use of different data sources for flood prediction by data integration. For example, studies show that the crowdsourced data is biased toward less vulnerable populations and the areas with higher population^80^, therefore, solely relying on these data poses great biases on the predictions while including flood sensor data that are somehow evenly distributed across the county chiefly based on the flood exposure and without consideration of population, reduces the bias in the model prediction.

In addition to flood reports and social media activities, recent studies show that telemetry-based human activity fluctuations, which is registered by the concentration of aggregated usage of cellphone users in specific areas can signal flood inundation or other disaster-related impacts^82,83^. To incorporate information regarding human activity in our flood nowcasting model, we obtained digital traces of human activities for the study timeframe from Mapbox. We chose Mapbox as the source of the telemetry data due to its ability to collect temporal and spatial telemetry-based human activity with a proper level of aggregation. Human activity is collected, aggregated, and normalized by Mapbox based on the geography information updates of locations of users’ devices (such as cell phones) from applications that use Mapbox Software Development Kit (SDK). Human activity here is calculated as the density of the usage of cellphone users in specific areas that are recorded, aggregated, and anonymized by Mapbox SDK globally contributing to live location updates. (The data is gathered from app developers who access Mapbox data through the SDK. Mapbox records locations of users of the maps service.) Mapbox provided a 4-h temporal resolution as raw data. In terms of spatial resolution, tiles represent square geographic areas approximately 100 m per side, a size which varies depending on latitude. The more users located in a tile at time ${\varvec{t}}$, the greater the human activity index. Data might not exist for all spatial units, as data is derived from cell phone activity depending on the updates of the geography information of cell phone users. Moreover, to preserve privacy and the data aggregation process, traces are excluded from tiles with small numbers of users. The raw index of human activity is normalized. Normalization is compartmented separately by month and type of trace and yields a normalized activity index for each tile in each 4-h time period of human activity provided by Mapbox. The normalized values range between 0 and 1. We created time series of human activity by aggregating tiles into census tracts and averaging the activity indexes for all the tiles that fall into a census tract in a certain timestep. Thus, we used linear interpolation to aggregate indexes of human activity for each 30-min timestep as the time period considered for our model. Table 1 also provides a summary of dynamic features used for flood nowcasting in this study.

### ASTGCN model

#### Graph adjacency matrix

Considering that the graph represents an area, and each node represents a census tract, co-location of two census tracts can imply similarities between their state of flooding. Therefore, we considered the distance between census tracts as the major determinant of the weights in the adjacency matrix. In addition to physical distance, we considered static features that imply similarity in flooding status of two areas. In particular, we considered features that influence flooding status in a flood-prone urban area: (1) whether the area is inside the 100-year floodplain, (2) distance to the closest main streamflow, (3) distance to the outlet (Galveston Bay in our study area), (4) the watershed in which that the area is located, and (5) the land-use pattern. To include these static features in our adjacency matrix, we created a vector of size five for each census tract containing the static features and calculated the Euclidean distance similarity for each pair of census tracts. To combine the impact of static features and co-location dependency, we used the weighted average of the Euclidean distance similarity and the physical distance. Based on the early experiments on the model for tuning the weights for the adjacent matrix, we found that choosing 0.1 as the weight for Euclidean distance similarity and 0.9 as the weight for physical distance yields the best result.

#### Model architecture

We adopted the ASTGCN model architecture design from the model proposed by Guo et al.^33^ that was developed primarily as an attention-based graph convolutional network for forecasting traffic flow. The original model framework includes three independent input components and employs information fusion to consider different temporal properties of the traffic flow and to deal with the seasonality of the traffic data. In the case of flood nowcasting, however, there is often no seasonality in the temporal changes of major features—such as rainfall, stream elevation, and human activity—during the hazard period. Hence, we used a single input component in our architecture that consists of time series of three physics-based and three human-sensed dynamic features recorded for each node of the graph. Thus, given the six dynamic features, and $N$ nodes in the graph model of the area, all the features over the $T$ timesteps form $X={({x}_{1},{x}_{2},\dots ,{x}_{t},\dots ,{x}_{T})}^{T}$ as the input, where ${x}_{t}$ includes all the features for all the nodes at timestep $t$. Moreover, we used the percentage of inundated roads (determined based on INRIX traffic data) as the target variable and used ${y}_{t}^{i}$ to represent the flooding status of census tract $i$ at timestep $t$.

**Figure 4:**
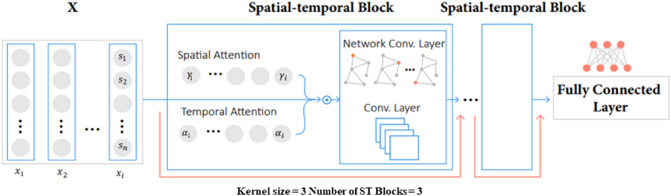
Model architecture, including model input, spatial–temporal blocks, attention layers, and the fully connected layer at the end.

As shown in Fig. 4, the ASTGCN model consists of spatial–temporal (ST) blocks and a fully connected layer. Each ST block consists of a spatial attention module and a temporal attention module that is followed by a spatial–temporal convolution module on the graph model. The attention modules are included to capture the spatial and temporal correlation of the dynamic heterogeneous input features in the nowcasting flood status. These modules enable the network to adjust the weights of the features and determine the pieces of data upon which the model needs to rely more heavily to have generate predictions. The output is then fed into the spatial–temporal convolution module that captures the dependencies between different nodes based on the adjacency matrix and the time series of input features. The model includes $L$ ST blocks, where the input for $(l+1)$th block is:

1 $$X_{{}}^{l} = \left( {x_{1} ,x_{2} ,x_{3} , \ldots ,x_{{T_{l} }} } \right) \in {\mathbb{R}}^{{N \times C_{l} \times \tau_{l} }}$$

where ${C}_{l}$ denotes features of the input data in the $(l+1)$th layer, ${\tau }_{l}$ denotes the length of the temporal dimension in the $l$th layer, which for $l = 1$, equals $T$. The spatial attention is then determined as follows:

2 $$SAtt = P_{s} \cdot \sigma \left( {X^{l} W_{1} } \right)W_{2} \left( {W_{3} X^{l} } \right)^{T} + b_{s}$$

where ${P}_{s}$ and ${b}_{s}$ are $N\times N$ learnable parameters, and ${{W}_{1}}_{{C}_{l}},{{W}_{2}}_{{C}_{l}\times {\tau }_{l}},$ and ${{W}_{3}}_{{C}_{l}}$ are also learnable parameters that are fed into sigmoid function $\sigma$ as the activation function. Similarly, the temporal attention module captures the strength of information between two timesteps $i$ and $j$. After processing at the attention modules, the data becomes more valuable for the convolution layer as it extracts and captures both dynamic spatial and temporal dependencies. The data is then fed into the spatial–temporal convolution module, which also has spatial and temporal dimensions. For applying convolution of the network structure, Guo et al.^33^ used the spectral graph theory, and for each timestep, graph convolutions operate on the graph to extract correlation in the spatial dimension based on the developed adjacency matrix. Given $D$ as the degree matrix and $A$ as the adjacency matrix, Laplacian matrix ($L$) is defined as follows:

3 $$L = D - A$$

The normalized form of the Laplacian matrix is used to apply convolution on the graph as follows:

4 $$g_{\theta } *_{G} x = g_{\theta } \left( L \right)x = g_{\theta } \left( {U{\Lambda }U^{T} } \right)x = Ug_{\theta } \left( {\Lambda } \right)U^{T} x$$

where ${*}_{G}$ operates a convolution on the graph $G$ given the signal $x\, and \,U$ is Fourier basis. Guo et al.^33^ adopt a Chbyshev polynomial to approximate the eigenvalue decomposition on the Laplacian matrix and get the neighborhood of 0 to $k-1$-order of each node by ${g}_{\theta }$ as follows:

5 $$g_{\theta } *_{G} x = \mathop \sum \limits_{k = 0}^{K - 1} \theta_{k} T_{k} \left( {\tilde{L}} \right)x$$

where $\theta$ consist of $K$ polynomial coefficients, ${T}_{k}\left(x\right)=2x{T}_{k-1}\left(x\right)-{T}_{k-2}(x)$ and $\widetilde{L}$ is determined as follows:

6 $$\tilde{L} = \frac{2}{{\lambda_{max} }}L - I_{N}$$

and ${\lambda }_{max}$ is the maximum eigenvalue of the Laplacian matrix. The Hadamard product of ${T}_{k}\left(\widetilde{L}\right)$ and ${SAtt}^{^{\prime}}$ is used in the approximation to include the effect of the spatial attention. Doing so, we can perform required number of filters for each node at each timestep and ensure that the neighboring information has been captured in the spatial dimension. Next, we use the similar standard temporal convolution to update the information based on the past timesteps; for the $l$th layer, we have:

7 $$X^{l} = ReLU\left( {{\Phi }*\left( {ReLU\left( {g_{\theta } *_{G} X^{l - 1} } \right)} \right)} \right)$$

where * represents standard convolution, $\Phi$ parameters of temporal kernel, and $ReLU$ is the rectified linear unit activation function. The model in this study includes three appended ST blocks that are stacked to a fully connected layer that uses a softmax activation function for classifying the dependent variable, flood status.

#### Model evaluation

The original ASTGCN model that has been adopted by this study is a regression model. However, based on the nature of this study, we transformed the task to classification. The reason for transferring the model from regression to classification is mainly the imbalance in the predictor variable (i.e., the road data). As a common issue in flood prediction task, the data is often imbalance toward non-flooded values. Our experiments on both regression and classification tasks showed that splitting flood extent can reduce the imbalance in the data and better capture the flooding in the model prediction. In addition, interpretability of the performance of the model in classification task is the other reason. Although metrics such as MAE and RMSE can be compared for different models in the regression task, the comparison cannot provide proper insight on the model performance on failure to capture flooding (recall) and incorrect detection of flood (precision), which are very important to understand the performance of predictive models in rare event predictions.

We employed various classification metrics which can capture the performance of the model on the imbalance data. Accuracy, precision, recall, and F1 score are used for the case that the target variable is a categorical variable capturing the status of flooding. Our target variable has three different classes, thus we employed macro precision, recall, F1 score, and accuracy as model evaluation parameters to highlight the performance of the model on the minority class.

It should be noted that while this study adopted the ASTGCN model structure, it provides various modifications and adjustments to make the model suit for the problem in this study versus the traffic prediction task in the initial version of the ASTGCN model proposed by^33^. These adjustments have been performed to ensure that the model proposed by Guo et al.^33^ is properly adopted for the purpose of this study^84^. These adjustments include (1) transforming the model structure to perform a classification task instead of the regression task in the original model. This has been done by modifying the activation function and using evaluation metrics fitted for classification task, (2) varying the number of layers to yield the optimal model performance by doing multiple experiments, (3) modifying the method for determining the weights for the formation of the graph adjacency matrix based on features other than the distance to capture similarity in flood-related features, and (4) stacking and feeding various curated data into the model (versus feeding the model by single feature aggregated in different time periods in the original model).

## Results

### Study context

As one of the most flood-prone areas in the United States, Harris County has experienced several devastating floods since the latter half of the twentieth century. Notably, Hurricane Harvey, as a Category 4 hurricane, made landfall in Texas on August 25, 2017. Hurricane Harvey led to a catastrophic flood that necessitated 100,000 rescue requests in the week following its landfall in Harris County, as well as damage to 80,000 structures^75^. Contained within Harris County’s 1777 square miles are 22 primary watersheds. Detailed information regarding individual watersheds can be found at the Harris County Flood Control District website^85^. Each watershed has independent flooding management issues. Some of them merge and drain into one of the major creeks or bayous, but ultimately, all stormwater drains into Galveston Bay. We defined our study timeline from August 25, 2017, to September 3, 2017, and we collected the sets of data required for the flood nowcasting model for 787 census tracts in Harris County.

### Implementation details

In this study, we used data from August 25 to August 30, 2017, as our training set, and data from August 31, 2017, to September 3, 2017, as our test dataset. We used 30-min intervals to give us 288 timesteps for training the data and 192 timesteps for testing the model. We split the data in a way that both training and testing sets capture portions of flood propagation and recession due to Hurricane Harvey. There were two main factors that governed the decision for data split. First, to perform proper prediction, both training and testing datasets need to include sufficient (and ideally close) ratio between the number of observations in each classes of the response variable. However, the fact is that the flooding mainly occurs in the specific timeframe and it is challenging to split the data in a way that both datasets include a reasonable case of flooding. To deal with this issue, we plotted the flood status time series to understand what threshold would best divide the data into train and test set that capture sufficient flood cases. It leads to selecting the existing train/test datasets in which the ratio of the test dataset is slightly larger than the convention. Besides, the other consideration is that the model needs to see sufficient number of data points to perform proper prediction based on the time series of the observed data. therefore, the test set cannot be as small as the conventional models employ (e.g., 10% of the entire dataset). Accordingly, all the dynamic features were extracted and underwent data preprocessing required for feeding into the model. In cases that the dynamic features were not available in the time units of the study, linear interpolation and extrapolation were used to extract the required values for the missing timesteps. We categorized flooding statuses into three classes: in each timestep, census tracts with fewer than 1% of roads flooded are considered as “no flood,” census tracts with 1%–10% of roads flooded are considered as “moderate flood, and census tracts with more than 10% roads flooded are considered as “severe flood.” The selection of the ratio for determining classes of flood extent was primarily done by a combination of testing different ratios and authors’ judgement. In fact, we plotted the different ratios to see which ratio can better reflect the flood extent in the area in accordance with other ground truths such as flood maps during Hurricane Harvey. In should be noted that since the ground truth in this study captures road/street flooding, it does not perfectly match with flood maps since not all flood inundations cause street flooding. however, the overall comparison can be made using the flood maps and the historical information regarding the flood impact during 2017 Hurricane Harvey in Harris County.

In this case, the model solves a classification problem in which the objective is to minimize the misclassified samples. We performed hyperparameter tuning by focusing on the learning rate and dropout rate to select the model with the best performance.

### Model performance and comparison

Along with model implementation and to better evaluate the model performance, we used different state-of-the-art models against which ton compare the performance of the ASTGCN model. Moreover, we examined the extent to which the integration of human-sensed data can improve the performance of a model that relies solely on physics-based data for flood nowcasting. To this end, we ran four different experiments. First, we ran the model on the attention-based spatial–temporal graph convolution network model fed by physics-based data (model 1). Next, we employed the same ASTGCN model and employed both physics-based and human-sensed features as input (model 2). To assess the impact of the attention mechanism on the model performance, we used a relatively similar spatial–temporal graph convolutional network (STGCN) model (model 3) adopted from Yu et al.^86^. Finally, we used a long-short term memory (LSTM) model (model 4) as the baseline for model performance comparison.

Table 2 shows the performance of the models in terms of precision, recall, F1 score, and model accuracy. Comparing the performance of graph-based models (models 1, 2, and 3) with the LSTM model, we can see that the graph-based models show significantly better performance in terms of precision, recall, and F1 score, while all the models have proper accuracy. The poor performance of the LSTM model in macro precision, recall, and F1 score shows that the model is unable to classify minority classes (i.e., flooded areas), which indicates that the model cannot provide insight for flood nowcasting. Comparing the performance of graph-based models, the STGCN model demonstrates highest recall and accuracy. However, the precision is 9.28% lower than the model with the highest precision, model 2, which uses physics-based and human-sensed input. This considerable difference is also reflected in the F1 score. The implication is that model 3 properly captures flooded cases (high recall), which is particularly valuable for flood nowcasting since it ensures the majority of the flooded areas are captured; however, the downside is that it erroneously captures many non-flooded cases.

**Table 2:** Evaluation metrics for performance comparison of different models.

Criteria/Model	ASTGCN-I* (model 1)	ASTGCN-II** (model 2)	STGCN (model 3)	LSTM (model 4)
Precision	0.785	**0.808**	0.733	0.416
Recall	0.824	0.891	**0.906**	0.413
F1 score	0.802	**0.842**	0.819	0.414
Accuracy	0.975	0.979	**0.999**	0.981

Significant values are in [bold].

*ASTGCN with physic-based features.

**ASTGCN with physic-based and human-sensed features.

Finally, the comparison of model 1 and model 2 reveals valuable insights for flood nowcasting and risk prediction. As shown in Table 2, model 2 over-perform model 1 in major evaluation metrics, including precision, recall, and F1 score. Particularly, model 2 yields 2.92% higher precision, 8.13% higher recall, and a 4.99% higher F1 score. Therefore, it can be seen that the use of human-sensed features as the supplement to physics-based input for flood nowcasting in the graph-based model significantly improves the predictive performance of the model. This finding shows the benefit of using heterogeneous community data and integrating different dynamic features for flood nowcasting. It reinforces the need for developing pipelines for collecting, preprocessing and integrating human-sensed data that becomes available during a flood event to improve awareness.

**Figure 5:**
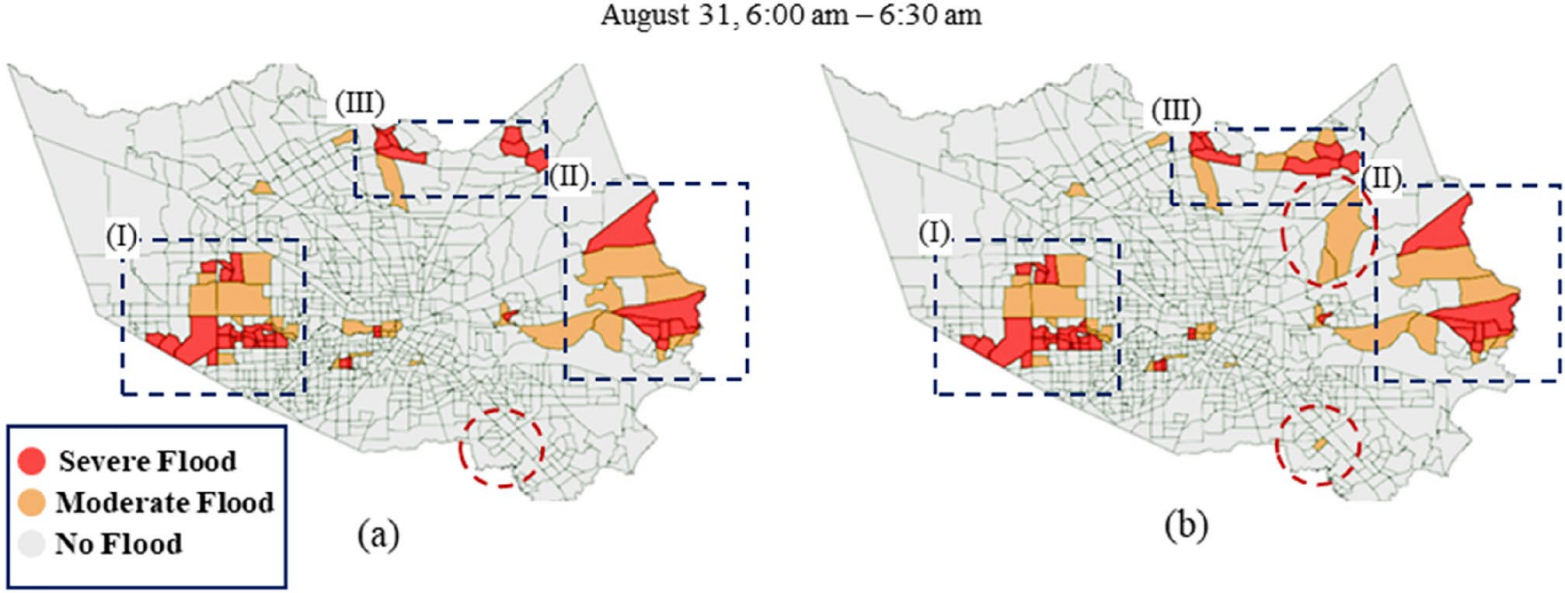
An example of model overall predictive performance (August 31, 6:00 a.m.–6:30 a.m.); (**a**) ground truth versus (**b**) model prediction.

**Figure 6:**
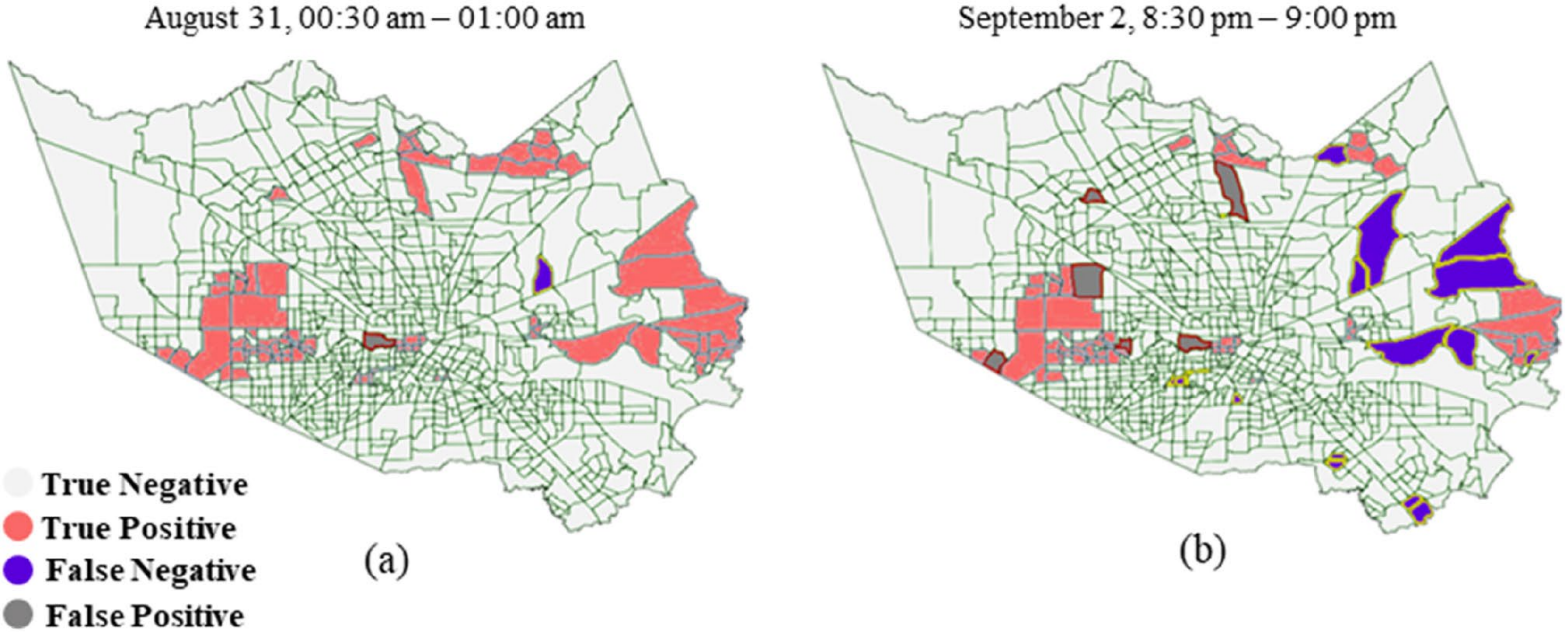
An example of model flood nowcasting performance; (**a**) a proper prediction (August 31, 00:30 a.m.–01:00 a.m.) versus (**b**) model prediction with more misclassification (September 2, 8:30 p.m.–9:00 p.m.).

Figure 5 shows an instance of the prediction performance for model 2. As can be seen in boxes (I), (II), and (III), the model performed well in the case of the clusters of flooded areas, although in some cases (box (II)), there are misclassified regions. These region errors might indicate the impact of capturing the spatial dependency on the predictive performance that enables the model to identify the inundation hot spots and aid decision-makers to detect regions that need to be prioritized for emergency response in near future. On the other hand, as we can see in the red circles in Fig. 5b, particular areas that are not in the flooded clusters have been classified incorrectly. This result might indicate the need for more data, particularly human-sensed data, which can signal inundation of areas where flooding is difficult to detect by the co-location dependency. Figure 6 also shows two cases of flood nowcasting performance by model 2, which shows significant differences in predictive performance. As shown in Fig. 6a, the model has performed properly in identifying the majority of the flooded area; however, in considerable misclassified areas are evident in Fig. 6b. Considering that Fig. 6a shows a timestep close to the start time of the test set (timestep 2), while Fig. 6b is a timestep that captures the third day in the test set (timestep 136), it might be inferred that the model performance decays as the time passes, which can be addressed by updating the model during the flood event.

## Conclusions

A crucial step for effective and timely disaster response and recovery is situational awareness, how the situation is evolving, and how community actors and residents respond to the evolving situation^87^. In this regard, flood nowcasting plays a pivotal role in enhancing situational awareness by providing a realistic prediction of the areas at risk of flood inundation in near future. In this study, we adopted an attention-based spatial–temporal graph convolution network (ASTGCN) model for urban flood forecasting. The model employs both physics-based and human-sensed features, as well as static features that capture spatial dependency in terms of flood propagation. In the ASTGCN model, the attention mechanism enables automatically updating the importance of spatial and temporal dependencies for flood nowcasting, and the spatial and temporal convolutions extract the local dependencies in the model. We demonstrated the application of the model and compared its performance in the context of flooding following Hurricane Harvey in Harris County, Texas, in August 2017. The results indicate that, in general, the graph-based structure significantly improves prediction of flooded areas. For example, the model performs significantly better than the conventional long-term short-term memory models in terms of precision and recall, which are metrics of interest in prediction tasks using an imbalanced data set. Moreover, the attention mechanism improves the model precision and helps to capture the majority of flooded areas. The results also indicate that the ASTGCN model performs significantly better if it employs heterogeneous human-sensed data as a supplement of the physics-based data that traditionally used by hydraulic and hydrologic models. This finding is particularly significant since it demonstrates the promise of developing data pipelines for data fusion using physics-based data collected by flood gauges and sensors and data that is either generated by residents or captures the digital trace of residents’ activity.

The main contributions of this study are twofold: first, we adopted and tested a novel graph deep-learning model for urban flood nowcasting. Second, the study showed the value of leveraging human-sensed data to complement physical flood sensor data for observing flood status across a region to improve flood nowcasting. Through these contributions, this study advances the body of knowledge related smart flood resilience. The advances in a structured deep-learning model provide opportunities for employing model architectures that extract information from spatial and temporal dependencies^2^ and modules that extract information by putting more attention on the varying spatial and temporal features^44^. Moreover, the increasing availability of heterogeneous human activity data in near real-time calls for pipelines that leverage the information embedded in such data that can provide signals for urban flooding. The novel deep learning-modeling approaches and the availability of human-sensed data advance smart flood resilience by providing tools and pipelines that help people better respond and react to floods through enhanced predictive flood exposure and risk mapping before and during floods. This study, in particular, demonstrates the promise of integrating physics-based and human-sensed data into a graph-based deep-learning model that captures spatial and temporal dependencies for flood nowcasting. Also, this study showed the promise of data-driven models to complement physics-based H&H models for predictive flood monitoring and situational awareness.

It should be noted that the one of the main challenges in urban flood prediction studies is the scarcity of the required data in urban scale and limited data availability. The ideal case is to have data of various flood events to train model on specific events and test it on the other events to evaluate the model performance. However, this is only possible for various flood prediction studies that solely rely on sensor data^43^. When it comes to leverage emerging datasets such as human activity and crowdsourced data, some of the datasets (i.e., Mapbox and Twitter data) are currently available only for limited number of events (e.g., Hurricane Harvey in this study). Nevertheless, the availability of these datasets is increasing, and therefore, it is valuable to test the applicability of these data for future employment in larger scale. In fact, we aimed at using the existing datasets, acknowledging the abovementioned limitation in data scarcity, and aiming at limiting the impact of the data scarcity on the model performance by different techniques. While the model performance cannot guarantee the same model performance in the future events due to the data limitations, the comparison made by using various model structures shows the potential for the superior performance of the proposed framework (i.e., employing graph-based models and data integration to capture different flood signals).

Future studies can focus on developing techniques to reduce the computational demand of the existing models to make the use of these models more feasible for flood nowcasting once more data streams are fed into the model. Moreover, further studies can generalize the approach demonstrated in this paper by testing the model on other flood cases and utilizing other types of physics-based and human-sensed features as inputs. As mentioned earlier, one limitation of this study is that the model was tested in a single event and region, as the data used in this study was not available for historical events. As various physical sensor and human-sensed data become more available in future events, however, the model could be employed and tested in other events and contexts.

## Data Availability

All data were collected through a CCPA- and GDPR-compliant framework and utilized for research purposes. The data that support the findings of this study are available from Mapbox and INRIX, but restrictions apply to the availability of these data, which were used under license for the current study. The data can be accessed upon request by the data providers. Other data we use in this study are all publicly available.

## Code availability

The code that supports the findings of this study is available from the corresponding author upon request.
